# The Preeclamptic Environment Promotes the Activation of Transcription Factor Kappa B by P53/RSK1 Complex in a HTR8/SVneo Trophoblastic Cell Line

**DOI:** 10.3390/ijms221910200

**Published:** 2021-09-22

**Authors:** Agata Sakowicz, Michalina Bralewska, Tadeusz Pietrucha, Francesc Figueras, Dominika E. Habrowska-Górczyńska, Agnieszka W. Piastowska-Ciesielska, Agnieszka Gach, Bartosz Sakowicz, Magda Rybak-Krzyszkowska, Hubert Huras, Mariusz Grzesiak, Lidia Biesiada

**Affiliations:** 1 Department of Medical Biotechnology, Medical University of Lodz, 90-752 Lodz, Poland; michalina.bralewska@gmail.com (M.B.); t.pietrucha@gmail.com (T.P.); 2 Barcelona Center of Maternal-Fetal Medicine and Neonatology (Hospital Clinic and Hospital Sant Joan de Deu), IDIBAPS, University of Barcelona, 08036 Barcelona, Spain; FFIGUERA@clinic.cat; 3 Department of Cell Culture and Genomic Analysis, Medical University of Lodz, 90-752 Lodz, Poland; dominika.habrowska@umed.lodz.pl (D.E.H.-G.); agnieszka.piastowska@umed.lodz.pl (A.W.P.-C.); 4 Department of Genetics, Polish Mother’s Memorial Hospital-Research Institute in Lodz, 93-338 Lodz, Poland; agagach@o2.pl; 5 Department of Microelectronics and Computer Science, Lodz University of Technology, 93-005 Lodz, Poland; bartosz.sakowicz@gmail.com; 6 Department of Obstetrics and Perinatology, University Hospital in Krakow, 31-501 Krakow, Poland; rybaczka@interia.pl (M.R.-K.); huberthuras@wp.pl (H.H.); 7 Department of Perinatology, Obstetrics and Gynecology, Polish Mother’s Memorial Hospital-Research Institute in Lodz, 93-338 Lodz, Poland; mariusz.grzesiak@gmail.com; 8 Department of Obstetrics and Gynecology, Medical University of Lodz, 93-338 Lodz, Poland; 9 Department of Obstetrics and Gynecology, Polish Mother’s Memorial Hospital-Research Institute, 93-338 Lodz, Poland; bieslidia@o2.pl

**Keywords:** NFκB activation pathways, preeclampsia, p53/RSK1 complex, trophoblastic cell line

## Abstract

Preeclampsia is a pregnancy disorder associated with shallow placentation, forcing placental cells to live in hypoxic conditions. This activates the transcription factor kappa B (NFκB) in maternal and placental cells. Although the role of NFκB in preeclampsia is well documented, its mechanism of activation in trophoblastic cells has been never studied. This study investigates the mechanism of NFκB activation in a first trimester trophoblastic cell line (HTR8/SVneo) stimulated by a medium containing serum from preeclamptic (PE) or normotensive (C) women in hypoxic (2% O_2_) or normoxic (8% O_2_) conditions. The results indicate that in HTR8/SVneo cells, the most widely studied NFκB pathways, i.e., canonical, non-canonical and atypical, are downregulated in environment PE 2% O_2_ in comparison to C 8% O_2_. Therefore, other pathways may be responsible for NFκB activation. One such pathway depends on the activation of NFκB by the p53/RSK1 complex through its phosphorylation at Serine 536 (pNFκB Ser536). The data generated by our study show that inhibition of the p53/RSK1 pathway by p53-targeted siRNA results in a depletion of pNFκB Ser536 in the nucleus, but only in cells incubated with PE serum at 2% O_2_. Thus, the p53/RSK1 complex might play a critical role in the activation of NFκB in trophoblastic cells and preeclamptic placentas.

## 1. Introduction

The success of each pregnancy is determined by a complex set of processes, including trophoblast invasion into maternal decidua, remodeling of maternal spiral arteries, and the adaptation of the maternal immunological system to tolerate the foreign antigens of the developing fetus [1].

At the beginning of each pregnancy (<week 10 of gestation), the trophoblastic cell environment is characterized by low oxygen tension, creating a hypoxic environment of about 1–2% O_2_ [2]. This is critical for early embryo development and promotes the differentiation of trophoblastic cells and their invasion [3,4]. At about week 10 of pregnancy, the endovascular invasion of trophoblastic cells into the decidual segments of maternal spiral arteries increases oxygen tension in the intrervillous spaces [5], resulting in the establishment of normoxic conditions (6–8% O_2_) for the placental cells [4]. Preeclampsia is a serious complication affecting 5–8% of all pregnancies that is characterized by maternal and fetal morbidity or mortality [6]. It is recognized when the blood pressure of previously normotensive women increases to up to ≥140 mmHg systolic or ≥90 mmHg diastolic at, or after, 20 weeks’ gestation; additionally, the new onset of hypertension has to be complicated by at least one of the following symptoms: proteinuria, serum creatinine level >1 mg/dL, elevated transaminase levels, thrombocytopenia, haemolysis, neurological disorders or uteroplacental dysfunction (i.a., fetal growth restriction) [7,8]. Although the etiology of this phenomenon remains a mystery, it is known that both maternal and placental factors play a significant role in its development.

One of the main causes of preeclampsia is believed to be an inappropriate trophoblast invasion into maternal decidua at the early stage of pregnancy [6,9]. Abnormal, shallow placentation forces placental cells to develop in hypoxic conditions (about 1–2% of oxygen), provoking the development of oxidative stress [10]. This not only influences the metabolism, survival and behavior of the placental cells, but may also cause the generation of reactive oxygen species (ROS) in the maternal circulation [11]. Moreover, oxidative stress supports the development of both local and systemic chronic inflammation, which is regulated by transcription factors, including nuclear factor kappa B (NFĸB) [12,13].

NFĸB activation is known to occur through various pathways; however, the canonical, the non-canonical and the atypical pathway are the most well known. In the canonical pathway, NFĸB is activated by a high-molecular-weight complex containing two following catalytic subunits: Inhibitor of Nuclear Factor Kappa B Kinase Subunit Alpha (IKKα) and Beta (IKKβ) and a scaffolding subunit i.e., Inhibitor of Nuclear Factor Kappa B Kinase Subunit Gamma (IKKγ). This complex is responsible for the phosphorylation of both NFĸB and its inhibitor, i.e., NF-Kappa B Inhibitor Alpha (IĸBα) or NF-Kappa B Inhibitor Beta (IĸBβ). Following phosphorylation, the inhibitor undergoes ubiquitination and degradation in the proteasome, allowing for the translocation of free factor ĸB into the nucleus [14]. In the non-canonical pathway, only homodimer IKKα is responsible for NFĸB activation. Finally, in the atypical pathway, casein kinase 2 (CK2), consisting of CK2α and CK2β subunits, participates in the degradation of NFĸB inhibitor. The activation of NFκB and its transcriptional activity are governed by its phosphorylation at Serine 536 by the IKKα and IKKβ kinases. CK2 is generally linked with phosphorylation at Serine 527; however, some studies report that inhibition of CK2 suppress NFκB phosphorylation at Serine 536 [15,16,17]. 

High NFĸB expression and activation are observed in both maternal cells and preeclamptic placentas [18]. However, while NFĸB is known to enhance the expression of genes implicated in the pathomechanism of preeclampsia (i.e., TNFα, IL-1 or IL-6), its mechanism of regulation in placental cells remains unclear.

This study investigates the process of NFĸB activation in a trophoblastic cell line under stimulation by maternal serum obtained from preeclamptic or normotensive women. To make the in vitro environment closer to that prevailing in the maternal womb, the process of NFκB activation was analyzed under hypoxia (2% O_2_) and normoxia (8% O_2_). As the process of preeclampsia begins at the early stage of the pregnancy, the study employed a human first trimester trophoblast cell line (HTR8/SVneo).

## 2. Results

### 2.1. Both the Maternal Serum Type and Oxygen Tension Influence the Level and Activity of Kappa B in the HTR8/SVneo Cells

**Figure 1:**
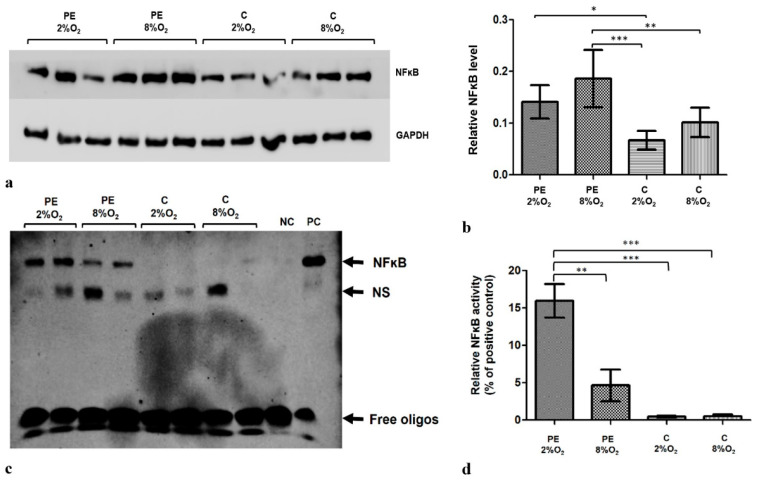
The differences in NFκB level and activity between HTR8/SVneo cells incubated in hypoxia and normoxia with medium containing 1% serum from preeclamptic (PE) or normotensive women (C). (**a**) Western blot membrane showing bands for NFκB and the control GAPDH protein; (**b**) NFκB level relative to GAPDH and Tubulin levels; the *p*-value was calculated by ANOVA with the Bonferroni post hoc test; * *p* < 0.05; ** *p* < 0.01; *** *p* < 0.001. Data are presented as mean ± SEM; (*n* = 3 experiments) (**c**) EMSA results. (**d**) EMSA results present as the relative NFκB activity calculated as percent of positive EMSA control; the *p*-value was calculated by the ANOVA with the Bonferroni post hoc test; ** *p* < 0.01; *** *p* < 0.001. Data are presented as mean and ± SEM; (*n* = 3 experiments). Abbreviations: PE 2% O_2_—hypoxia (2% O_2_), medium containing 1% serum from preeclamptic mothers; PE 8% O_2_—normoxia (8% O_2_), 1% serum from preeclamptic mothers; C 2% O_2_—hypoxia (2% O_2_), 1% serum from normotensive pregnant women; C 8% O_2_—normoxia (8% O_2_), 1% serum from normotensive pregnant women; NC—negative control; PC—positive control; NS—nonspecific band.

HTR8/SVneo cells demonstrate lower NFĸB concentration in hypoxia than in normoxia (8% O_2_). Moreover, the level of factor ĸB in the cells is influenced by the addition of 1% of serum from preeclamptic mothers to cell culture medium (Figure 1a,b). Of the tested experimental variants, the highest NFĸB level was observed in cells kept in medium with preeclamptic maternal serum in 8% O_2_. 

The EMSA results indicate that the addition of serum from preeclamptic pregnant women to the HTR8/SVneo cell culture influences NFĸB activity (Figure 1c); the strongest reaction was observed for cells incubated with PE 2% O_2_ (Figure 1d). As NFĸB level and activity is known to be elevated in preeclampsia, our findings indicate that the components included in maternal serum may play a role in its development.

### 2.2. The Canonical, Non-Canonical and Atypical NFκB Pathways Are Influenced by the Oxygen Tension and Maternal Serum

Both oxygen level and maternal serum type influence the level of the studied activators (IKKα, IKKβ, IKKγ, CK2α) and inhibitors (IκBα and IκBβ) in the studied cells (Supplementary Materials Figure S1a–g). 

The levels of IKKα and CK2α demonstrate a common trendline for results obtained between stimulation regimens. The cells kept in 8% oxygen tension present higher levels of IKKα and CK2α proteins in comparison to the cells stimulated by the same maternal serum in 2% O_2_; however, the results are not always statistically significant. Interestingly, the cells cultured in preeclamptic conditions, i.e., PE 2% O_2_, demonstrated significantly lower levels of IKKα and CK2α in comparison to cells living in normoxia with serum obtained from normotensive pregnant women (*p* < 0.001 Figure S1a,b). 

IKKγ protein expression was influenced by oxygen concentration. Cells living in normoxia demonstrated significantly lower levels of the studied protein than cells stimulated by the same serum in 2% O_2_ (i.e., IKKγ in PE 8% O_2_ < PE 2% O_2_ and in C 8% O_2_ < C 2% O_2_; *p* < 0.001 and *p* = 0.012, respectively). Under normoxia, maternal serum only had a significant effect at the 8% oxygen concentration: a significant lower level of IKKγ was observed in PE 8% O_2_ in comparison to C 8% O_2_ (*p* = 0.041) (Figure S1c).

Interestingly, oxygen tension does not seem to play a significant role in the regulation of IKKβ protein expression, with no significant differences observed between regimens. However, maternal serum factors appear to have a strong impact on IKKβ protein level in trophoblastic cells in 2% oxygen tension, with IKKβ level in PE 2% O_2_ being significantly lower than in C 2% O_2_; *p* = 0.023 (Figure S1d). 

Regarding the studied NFκB inhibitors (IκBα and IκBβ), the interaction between oxygen tension and maternal serum appears to regulate their expression; significant differences in IκBα and IκBβ concentrations were observed between cells incubated with PE 2% O_2_ and C 8% O_2_ (*p* < 0.001 and *p* = 0.004, respectively) (Figure S1e,f).

**Figure 2:**
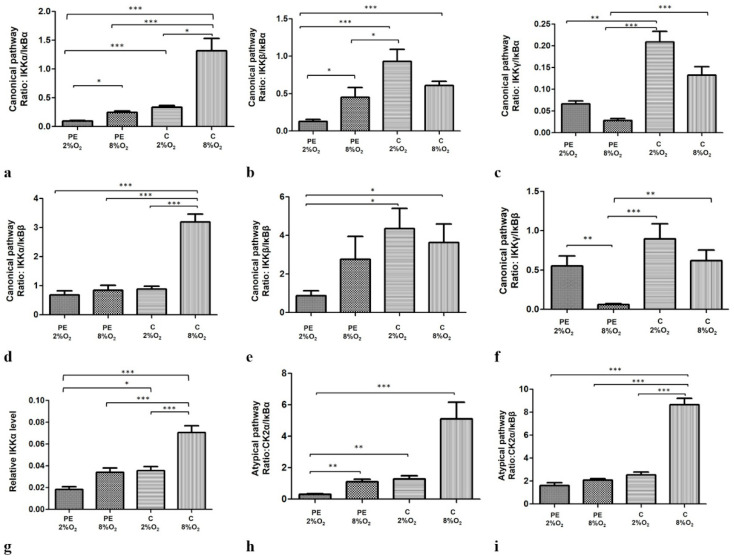
Comparison of the efficiency of the canonical (**a**–**f**), non-canonical (**g**) and atypical (**h**,**i**) NFκB activation pathways. Each profile was calculated based on the ratio of one of the activators to one of the NFĸB inhibitors in the studied pathway; however, the non-canonical pathway was calculated based only on IKKα protein level. The data are presented as mean ± SEM (*n* = 3 experiments), *p*-value calculated by ANOVA with the Bonferroni post hoc test or Kruskal–Wallis with Dunn’s post hoc test, depending the data distribution. * *p* < 0.05; ** *p* < 0.01, *** *p* < 0.001.

Moreover, our findings indicate that both oxygen tension and maternal serum, either separately or together, significantly modify the profiles of the canonical, non-canonical and atypical NFκB activation pathways. The efficiency profiles of the studied pathways were calculated based on the ratio of one of the activators to one of the inhibitors thus: IKKα/IĸBα, IKKα/IĸBβ, IKKβ/IĸBα, IKKβ/IĸBβ, IKKγ/IĸBα and IKKγ/IĸBβ for the canonical pathway and CK2α/IκBα and CK2α/IκBβ for the atypical pathway. The profile of the non-canonical pathway is only based on IKKα level, as this protein directly activates kappa B. Interestingly, all calculated ratios, except IKKγ/IĸBα and IKKγ/IĸBβ, were significant depleted for cells cultured in PE 2% O_2_ in comparison to these living in C 8% O_2_. As IKKγ cooperates with IKKα and IKKβ in the activation of NFĸB through the canonical pathway, and the calculated ratios for IKKα/IĸBα, IKKβ/IĸBα, IKKα/IĸBβ and IKKβ/IĸBβ were significantly lower for cells seeded in PE 2% O_2_ compared to C 8% O_2_, it is possible that the canonical pathway is downregulated in a preeclamptic environment. The results suggest that the canonical, non-canonical and atypical pathways do not play a significant role in the activation of NFκB in preeclampsia. The results of the calculated ratios for the three pathways are presented in Figure 2a–i.

### 2.3. The Distribution of p53/RSK1 Complex in HTR8/SVneo Cells Depends on Oxygen Tension; the Serum of Preeclamptic Women Favours the Maintenance of p53/RSK1complex Formation in Hypoxia

**Figure 3:**
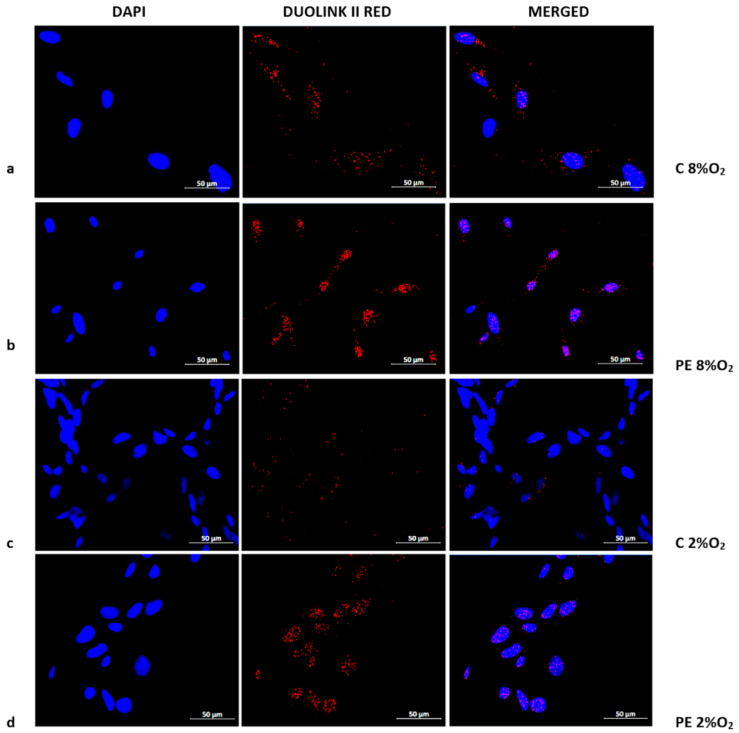
Duolink II PLA assay results for HTR8/SVneo cells cultured in normoxic and hypoxic conditions in medium containing 1% serum from preeclamptic (**b**,**d**) or normotensive (**a**,**c**) women. Each p53/RSK1 complex in HTR8/SVneo cells is visualised as an individual fluorescence red spot, nuclei were labelled with DAPI. Red spot – complex of p53/RSK1; blue spot – nucleus of cell. Magnification 40×; scale bar 50 µm.

The presence of the Tumor Protein p53/Ribosomal protein S6 kinase alpha-1 (p53/RSK1) complex was found to be characteristic of normoxia (8% O_2_), irrespective of normotensive or preeclamptic serum (Figure 3a,b). Hypoxia appears to prevent the formation of p53/RSK1; however, while supplementation with serum from preeclamptic mothers may sustain its formation, serum from normotensive women results in a very weak p53/RSK1 reaction in 2% oxygen. (Figure 3c,d).

### 2.4. The Depletion of IKKα by Targeted siRNA Downregulates the Phosphorylated Fraction of NFĸB at Serine 536 (pNFκB) in the Nucleus of Cells Stimulated by Serum from Normotensive Women

**Figure 4:**
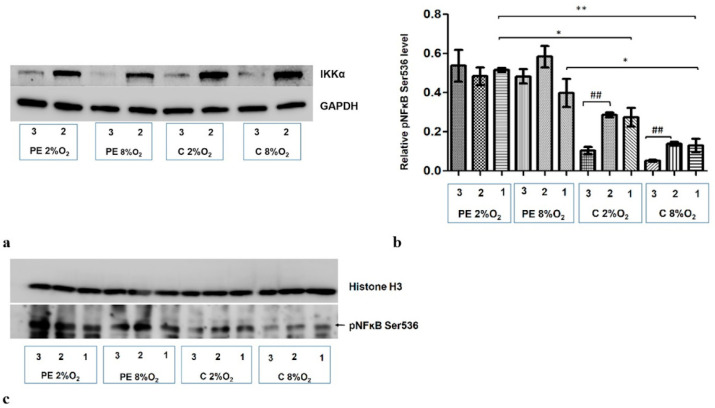
Western blot results. (**a**) inhibition of IKKα by targeted siRNA; Western blot results for the cytoplasmic fraction (**b**) the differences in relative pNFĸB Ser536 fraction to Histone H3 in nucleus of HTR8/SVneo cells. Cells were cultured for 96 h in hypoxic or normoxic conditions with medium containing 1% serum from preeclamptic or normotensive women. For the first 48 h, the cells were incubated only with medium containing maternal serum. After 48 h and 72 h, the cell medium was supplemented with the following factors: none, i.e., cells without siRNA (1), non-targeting siRNA (2) and siRNA targeted against mRNA coding for IKKα (3). The relative level of pNFĸB Ser536 compared to Histone H3 in the nuclear fraction was analyzed. Values are presented as mean ± SEM; (*n* = 3 experiments). The difference in pNFĸB Ser536 level between cells stimulated by negative and targeted siRNA was compared using Student’s *t*-test; ^##^ *p* < 0.01. The difference in pNFĸB Ser536 level between the four culture types (i.e., oxygen tension and serum type) which were not stimulated with siRNA were analyzed by ANOVA with the Bonferroni post hoc test; * *p* < 0.05, ** *p* < 0.01. (**c**) Western blot results for the nuclear fraction.

A significant reduction in IKKα level was observed following administration of targeted siRNA (Figure 4a). As IKKα is known as NFĸB S536 kinase, the nuclear fraction was analyzed for phosphorylation at the Serine 536 fraction of kappa B after the administration of both non-targeted and targeted siRNA (IKKα siRNA). In cells stimulated with serum from normotensive women, a significant reduction of pNFκB was observed in the nuclear fraction in both normoxic and hypoxic conditions after inhibition of IKKα. No significant change in pNFĸB S536 was observed following the addition of IKKα siRNA to the culture with preeclamptic maternal serum (Figure 4b,c). 

The results also indicate that maternal serum (i.e., PE or C) without addition of siRNA also influences the accumulation of Ser536 kappa B in the nucleus; preeclamptic serum favours this process (Figure 4b,c). 

Because pNFκB nuclear level did not change in cells cultured with serum from preeclamptic mothers after silencing of IKKα, it may suspect that the canonical or non-canonical pathways do not play a critical role in the activation of kappa B in trophoblastic cells in a preeclamptic environment.

### 2.5. Treatment with MG-132, an Inhibitor of Proteasome Activity, Does Not Influence the Accumulation of Phosphorylated IĸBα Protein in the Cytoplasmic Fraction of Cells Incubated in Hypoxia with Serum from Preeclamptic Mothers

**Figure 5:**
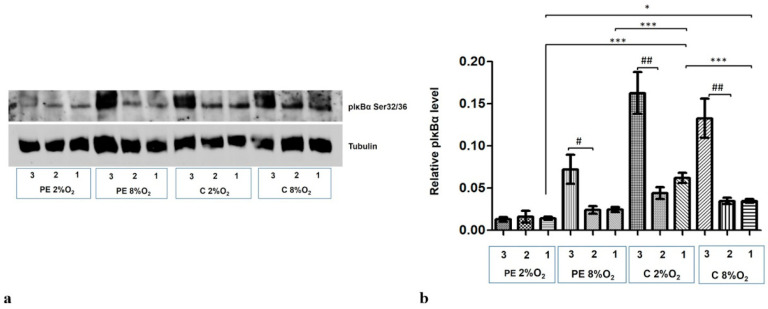
Western blot results for pIκBα Ser32/36 in the cytoplasmic fraction after MG-132 stimulation. The cells were cultured for 96 h in hypoxic or normoxic conditions with medium containing 1% serum from preeclamptic or normotensive women. For the last three hours, the culture medium was supplemented with the following factors: MG-132 (3), DMSO as the MG-132 vehicle (2) or none, i.e., cells incubated only with medium containing maternal serum without MG-132 or DMSO addition (1). (**a**) The Western blot membrane with pIκBα and Tubulin as a loading control (**b**) the analysis of Western blot results. Values are presented as mean ± SEM; (*n* = 3 experiments). The differences in pIκBα level between cells with DMSO and MG-132 were analyzed by Student’s *t*-test, # *p* < 0.05, ## *p* < 0.01. The difference in pIĸBα level for cells not stimulated by DMSO or MG-132 but grown in hypoxia/normoxia and control/preeclamptic serum were analysed by ANOVA with the Bonferroni post hoc test; * *p* < 0.05, *** *p* < 0.001.

The level of phosphorylation at the Serine 32/36 fraction of IĸBα (pIκBα) increased dramatically in the cytoplasmic fraction following the addition of 10 µM MG-132 to the cells for the last three hours of the 96-h stimulation. Only the PE 2% O_2_ cells did not demonstrate any elevation in the pIĸBα fraction (Figure 5a,b).

Western blot results also indicate that maternal serum influences the regulation of pIĸBα level in cytoplasm. The cytoplasmic fraction of cells incubated in PE 8% O_2_ and PE 2% O_2_ demonstrated significantly less pIĸBα than those stimulated by serum from normotensive women. 

As the pIκBα cytoplasmic level is lower in the preeclamptic environment, and its level did not increase in the PE 2% O_2_ group following MG-132 stimulation, it is possible that both the canonical and atypical pathways of NFĸB activation do not play a significant role in preeclampsia.

### 2.6. The p53 Protein Is Implicated in NFκB Activation in HTR8/SVneo Cells Incubated in Hypoxic Conditions with Medium Containing 1% of Serum from Preeclamptic Mothers

**Figure 6:**
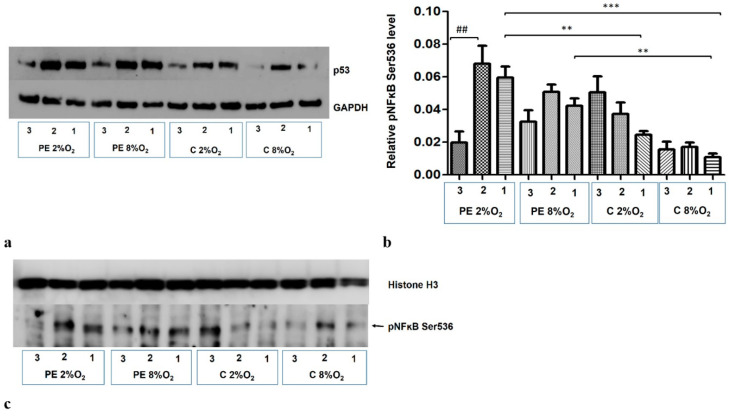
Western blot results. (**a**) inhibition of p53 by its targeted siRNA; Western blot results for the cytoplasmic fraction, (**b**) the differences in relative level of pNFĸB Ser536 to Histone H3 in nucleus of HTR8/SVneo cells. Cells were cultured for 96 h in hypoxic or normoxic conditions with medium containing 1% serum from preeclamptic or normotensive women. For the first 48 h, the cells were incubated only with medium containing the maternal serum. After 48 h and 72 h, the cell medium was supplemented with the following factors: none i.e., cells without siRNA (1), non-targeting siRNA (2) and targeted siRNA against mRNA coding for p53 (3). The relative level of pNFĸB Ser536 to Histone H3 in the nucleus was analyzed. Values are presented as mean ± SEM; (*n* = 3experiments). The difference in pNFĸB Ser536 level between cells stimulated by negative and targeted siRNA was calculated by the Student’s *t*-test; ## *p* < 0.01. The difference in pNFĸB Ser536 level between the four culture types (i.e., oxygen tension and serum type) which were not stimulated with siRNA were analyzed by ANOVA with the Bonferroni post hoc test; ** *p* < 0.01, *** *p* < 0.001. (**c**) Western blot results for nuclear fraction containing pNFĸB Ser536 and Histone H3, as a loading control.

The addition of Silencer Select p53 siRNA to the medium for the last 48 h of the 96-h cell culture reduces the p53 protein level in the cells (Figure 6a). The p53/RSK1 complex is known to activate NFκB by its phosphorylation at Ser536, independent of the cytoplasmic degradation of the kappa B inhibitor [19]. As the Duolink II PLA assay revealed that p53/RSK1 is detectable in all four variants of stimulation, the next stage of our study investigated whether it has an impact on the activation of NFκB in the studied environments. It was found that knockdown of p53 by its targeted siRNA significantly reduces the level of pNFκB Ser536 in the nucleus of HTR8/SVneo cells incubated in PE 2% O_2_; however, the pNFκB fraction does not change significantly in other three environments (Figure 6b,c). This suggests that factors existing in the serum of preeclamptic women influence the activation of NFκB by the p53/RSK1 pathway in hypoxic conditions.

## 3. Discussion

Preeclampsia is characterized by elevated expression and activation of NFĸB [18]. However, the mechanism of NFĸB activation in placental cells remains unknown. This is the first study to provide information about the possible pathway regulating NFĸB activity in a trophoblastic cell line, i.e., HTR8/SVneo. The functional study indicated that the p53/RSK1 complex promotes NFĸB activity in trophoblastic cells living in a preeclamptic environment.

NFκB is a transcription factor required for the proper course of pregnancy. At the beginning of a pregnancy, the trophoblastic cells grow in an environment characterized by low oxygen tension, i.e., about 1–2% O_2_. These conditions promote proliferation and differentiation of trophoblastic cells into invasive cell subtypes [20]. Generally, the process of implantation requires an inflammatory environment, which is generated by both maternal and trophoblastic cells and it is related with elevated expression of inflammatory factors (i.e., IL-1, IL-6 IL-8 or TNFα) and molecules responsible for the degradation of the extracellular matrix (e.g., metalloproteinases; MMP2 and MMP9) [21,22,23,24]. Their expression is regulated by the active form of NFκB [25,26,27,28,29,30]. The necessity of NFκB in the process of implantation was confirmed on the animal models. The suppression of NFκB activation and its translocation into nucleus of cells in mice uterus was found to delay the process of implantation confirmed in animal models whereas the complete loss of NFκB activity in knockout mice results in death [31,32]. As implantation proceeds, the oxygen level in the environment increases; this steers trophoblast differentiation toward remodeling of the maternal spiral artery and allows the placenta to mature into an exchange organ [33,34]. The inflammatory reaction is gradually slowed, thus allowing the development of an immunosuppression stage needed to maintain pregnancy. This is accompanied by a reduction in the activity of NFκB [35].

In women demonstrating clinical symptoms of preeclampsia after week 20 of gestation, the process of trophoblast invasion into the maternal decidua is known to be abnormal. Although it is typical to observe a low oxygen level for trophoblastic cells up to week 10–12 of gestation, longer periods of hypoxia have an impact on their biology [20,24].

In such cases, extended hypoxia results in the generation of hypoxia-induced reactive oxygen species (ROS), which intensify NFκB activation in cells [36]. Preeclamptic placentas are known to demonstrate both higher expression and activation of NFκB [37,38,39]; however, the mechanism of its activation in trophoblastic cells remains a mystery.

Therefore, this study examined the mechanism of NFκB activation in the HTR8/SVneo trophoblastic cell line, i.e., first trimester cells, under normoxia (8% O_2_) and hypoxia (2% O_2_). The cultures were additionally incubated with medium supplemented by serum from either preeclamptic (PE) or normotensive (C) women; this provided an indication as to whether only oxygen, only some unidentified factors present in maternal serum, or both together might influence the regulation of NFκB activation.

It was observed that the NFκB level in HTR8/SVneo cells falls as oxygen tension reduces. However, the PE 2% O_2_ cells demonstrated higher, but not significant, level of NFκB in comparison to the PE C 8% O_2_. Surprisingly, the nuclear activity of NFκB varies significantly between PE and C type of stimulation. The EMSA results show that nuclear activity of NFκB strongly depends on some factors present in the maternal serum used in HTR8/SVneo cell culture, with higher activity being observed in cells inoculated with PE serum, both in hypoxia and normoxia. This is consistent with studies on placental samples, showing that the NFκB is activated nearly 10-fold more in preeclamptic placentas than these from normotensive pregnancies [38]. Interestingly, although NFκB was found to be augmented under PE, none of the NFκB activation pathways described elsewhere, i.e., canonical, non-canonical or atypical, seem to play a dominant role in this process. This observation is consistent with those of our previous study based on placental samples obtained from preeclamptic and normotensive women [18]. Although both studies indicate that the canonical, non-canonical and atypical pathways demonstrate lower efficiency in a preeclamptic environment, based on activator to inhibitor ratios, this is not sufficient evidence that these pathways are downregulated in preeclampsia. Therefore, further functional studies were warranted.

The canonical, non-canonical and atypical pathways were inhibited using IKKα- targeted siRNA or proteasome inhibitor (i.e., MG-132), and the nuclear level of the Serine 536- phosphorylated form of NFκB or cytoplasmic level of phosphorylated at Serine 32/36 form of IĸBα were examined.

It is known that IKKα takes part in the canonical and non-canonical activation of NFκB [40]. In addition, NFκB activation has been found to be suppressed by specific siRNA molecules inhibiting IKKα [41,42]. This kinase assists in the activation of NFκB by phosphorylating Serine 536, allowing for kappa B translocation into the nucleus [43]. In the present study, incubation with IKKα-targeted siRNA resulted in a significant depletion of NFκB Ser536 in the nuclear fraction for the C 8% O_2_ and C 2% O_2_ cultures in comparison to the same cultures supplemented with non-targeted siRNA. For the PE 8% O_2_ and PE 2% O_2_ cultures, concentration of NFκB Ser536 remained at a similar level for all variants, i.e., those incubated with IKKα targeted siRNA and those with non-targeted siRNA. This suggests that the downregulation of IKKα-dependent pathways of NFκB activation does not inhibit the activation of the kappa B factor in preeclampsia.

Interestingly, the atypical pathway seems to be also downregulated in preeclampsia. Studies suggest that the inhibition of proteolytic activity of 26S proteasome complex by MG-132 has a negative impact on the proteosomal degradation of the phosphorylated and ubiquitinated form of kappa B inhibitors [44,45]. In both the canonical and atypical pathways, the Serine at positions 32 and 36 of IĸBα protein is phosphorylated by the upstream kinases (i.e., IKKα/IKKβ complex or CK2) and then ubiquitinated [46,47], thus allowing it to be degraded by the proteasome complex. Following IĸBα degradation, a free phosphorylated form of NFκB is translocated into the nucleus [14]. Some studies indicate that inhibition of proteasome activity by MG-132 results in the accumulation of phosphorylated and ubiquitinated IĸBα in the cytoplasmic fraction [14,19]. In the present study, the addition of 10 µM MG-132 to the cell medium resulted in an increase in the level of IĸBα Ser32/36 in the cytoplasmic fraction for all stimulation variants except PE 2% O_2_; this suggests that simultaneous presence of hypoxia and factors existing in preeclamptic serum influences NFκB activation via a pathway independent of proteosomal IĸBα degradation. Hence, the canonical and atypical pathways seem to be downregulated in preeclamptic environment.

Previous studies indicate that cells employ a method of NFκB activation independent of IĸBα degradation e.g., oxidative stress induced the activation and nuclear translocation of NFκB without the IĸBα degradation in an endothelial cell line [48]. In this process, the p53/RSK1 complex plays a dominant role, being responsible for the phosphorylation of NFκB at Serine536 [19,49]. It is known that preeclampsia is related to the accumulation of ROS and inflammatory factors in maternal blood, and that the HTR8/SVneo cell line generates mitochondrial ROS in hypoxic condition [34,50,51,52]. Therefore, in the present study, the combination of maternal serum and hypoxic conditions certainly influenced the generation of oxidative stress. Interestingly, the Duolink II PLA analysis suggests that the p53/RSK1 complex is present in HTR8/SVneo cells, irrespective of the stimulation variant. While the cells incubated in normoxia demonstrated similar levels of p53/RSK1, higher levels were observed in PE 2% O_2_ than C 2% O_2_.

Interestingly, following treatment with p53-targeted siRNA, the level of NFκB Ser536 in the nuclear fraction was depleted only in the PE 2% O_2_ cells; no significant change was observed in the other variants. This suggests that the p53/RSK1 complex might play different roles in cells depending on the mode of stimulation. The literature data indicates that protein complex might play a different role in the same cell depending on the environment [53]. Our present findings do not indicate that p53/RSK1 plays any significant role in NFκB activation in the C 8% O_2_, C 2% O_2_ and PE 8% O_2_ cultures: only the PE 2% O_2_ cells demonstrated a significant nuclear depletion at Ser536-phosphorylated NFκB. This suggests that in cases of hypoxia supported by factors present in PE, NFκB activation occurs depending on the p53/RSK1 complex, and that it is possible that the same mechanism acts in preeclamptic placentas, which demonstrate more intense NFκB expression and activation.

The present study has some limitations. Firstly, a significant difference in maternal weight was observed between the preeclamptic and normotensive serum donors. Maternal overweight might predispose to the appearance of some factors in maternal blood which influence the result of the study. We believe that factors typical of preeclampsia dominate in pooled maternal serum. However, further studies based on overweight pregnant women without comorbidities are needed to check the reaction of the cells against factors characteristic of obesity. Second, the first trimester trophoblastic cell line was stimulated with serum obtained from mothers in the last trimester of pregnancy. As the cells may demonstrate different reactions to maternal serum obtained from the first, second and third trimesters, and hence differences in the NFĸB activation pathways, the further studies are warranted.

Our findings cast light on the mechanisms behind higher activation of kappa B factor in placental cells. This may help in defining more targeted preventive strategies. At present, no treatment exists for preeclampsia itself, and existing strategies are based on treating its clinical manifestations (hypertension and neurological symptoms) or on labor induction, at the cost of prematurity. Low-dose aspirin (ASA) can be prescribed in the first trimester to prevent preeclampsia in high-risk pregnancies [54]. ASA generates particles that act as “braking signals” in inflammation, i.a., by inhibition of nuclear translocation and connection of NFĸB with DNA [55,56]. However, ASA supplementation must be commenced early to be effective [54]. Hence, a better understanding of the modes of kappa B activation may open a window of opportunity to efficiently treat the root mechanisms of preeclampsia.

## 4. Materials and Methods

### 4.1. Collection of Blood Samples

Venous blood was collected into vacutainer EDTA tubes from preeclamptic and normotensive women before delivery. Preeclampsia was recognized after week 20 of gestation when the maternal blood pressure in previously normotensive woman increased over 140/90 mmHg (measured twice with an interval of at least six hours) and protein was observed in the maternal urine (>300 mg/24 or at least 2+ during a single urine test). Patients with late preeclampsia were qualified to the study at approximately the same week of gestation as the control group (*p* > 0.05). The control group included only healthy pregnant women, whose blood pressure did not exceed the value 130/80 mmHg.

In both groups, the patients met the following criteria: single pregnancy, no symptoms indicating chronic diseases or gestational diabetes mellitus or hypertension before pregnancy, BMI < 30kg/m^2^, no fetal chromosomal aberrations and no uterus activity, which signals delivery. The clinical characteristics of the women from whom the blood samples were obtained are presented in Supplementary Materials (Table S1). Informed consent was obtained from all women qualified to the study. The study was approved by the Medical University Ethics Committee and the protocol complies with the ethical principles of the Declaration of Helsinki.

After blood collection, the samples were immediately centrifuged (10 min at 2000× *g*) and the collected plasma was stored at −30 °C. Before the experiments, the samples were thawed and 20% CaCl_2_ was added to plasma at a ratio of 1:100 (CaCl_2_:plasma). Following incubation (one hour, 37 °C) to induce fibrin clot formation, the samples were centrifuged (10 min. 3000× *g*) to obtain the serum (recalcified plasma). Sera from preeclamptic or normotensive women were merged in the two independent tubes, sterilized (0.22 µm PES filter) and stored at −30 °C until use in culture medium.

### 4.2. Cell Culture

The immortalized first trimester trophoblast cell line HTR8/SVneo (CRL-3271) was obtained from the American Type Culture Collection (ATCC) and maintained at 37 °C and 5% CO_2_, in medium supplemented with 10% Fetal Bovine Serum (FBS) (Gibco-BRL, Waltham, MA, USA) and 1% penicillin/streptomycin (Invitrogen, Waltham, MA, USA). For the experiments, the cells were incubated with Ham’s F-12K Nutrient Mix medium (Gibco Invitrogen, Carlsbad, CA, USA) containing 1% penicillin/streptomycin and 10% FBS (Gibco-BRL, Waltham, MA, USA) for 24 h. Following this, they were incubated in stimulating medium, i.e., Ham’s F-12K medium containing 1% penicillin/streptomycin and 1% human serum from preeclamptic or normotensive women, for 96 h, with the medium being replaced each day. All experiments were performed at 5% CO_2_ and 37 °C under hypoxia (2% O_2_) or normoxia (8% O_2_). The experiments were conducted in InVivo2 Physiological Cell Culture Workstations (Baker Ruskinn, Sanford, ME, USA) to maintain constant conditions of oxygen and temperature. All experiments were repeated three times (*n* = 3 experiments).

### 4.3. Total Protein Extraction

Briefly, 0.25 × 10^6^ HTR8/SVneo cell suspensions were seeded in 6-well plates in medium with FBS and incubated in hypoxic or normoxic conditions for 24 h. Next, the stimulating medium was added. After 96-h incubation in stimulating medium, the cells were washed twice in dPBS (Gibco Invitrogen, Carlsbad, CA, USA) and treated with RIPA buffer (Sigma, St. Louis, MO, USA) with Halt™ Protease and Phosphatase Inhibitor Cocktail (Thermo Scientific, Waltham, MA, USA). The cells were transferred into chilled Eppendorfs and incubated on ice for 30 min. After centrifugation at 14,000 rpm for 10 min, the supernatant (total protein fraction) was collected and stored at −80 °C for further analysis. Total protein concentration was measured using the BCA method (Thermo Scientific, Waltham, MA, USA).

### 4.4. Cytoplasm and Nuclear Fraction Isolation

The 0.8 × 10^6^ cells were seeded in a 25 cm^2^ flask in medium with FBS and incubated in hypoxic or normoxic conditions for 24 h. Next, the stimulating medium was added. After 96-h incubation in stimulating medium, the cells were washed twice in dPBS (Gibco Invitrogen, Carlsbad, CA, USA) and cytoplasmic fraction buffer was added. Further processes were performed according to the manufacturers’ instructions (NE-PER Nuclear and Cytoplasmic Extraction Kit, Thermo Scientific, Waltham, MA, USA). The concentration of protein in each subfraction was measured by the BCA method (Thermo Scientific, Waltham, MA, USA).

### 4.5. Small Interfering RNA (siRNA) and MG-132 Transfection

Briefly, 0.8 × 10^6^ cell suspensions were seeded in 25 cm^2^ flasks in medium with FBS and incubated in hypoxic or normoxic conditions for 24 h. Next, the stimulating medium was added. During the 96-h incubation, the stimulating medium was supplemented with siRNA or MG-132 as follows: siRNA transfection. On the third and fourth day of incubation, the stimulating medium was supplemented with Opti-MEM™ I Reduced Serum Medium (Gibco Invitrogen, Carlsbad, CA, USA) containing Lipofectamine RNAiMAX (Thermo Scientific, Waltham, MA, USA) and 100 μM of Silencer^®^Select Validated siRNA for p53 (mix s605 and s607) or CHUK (Conserved Helix-loop-helix Ubiquitous Kinase) coding for IKKα (mix s3076 and s3077) or Ambion^®^ Silencer Negative Control #1. MG-132 stimulation. A total of 10 µM MG-132 (Calbiochem, Darmstadt, Germany) was added three hours before the end of the 96-h stimulation. The cytoplasm and nuclear fractions were then isolated. Each experiment was repeated three times.

### 4.6. Western Blot Assays

Western blot was used for analyses of total, nuclear and cytoplasm proteins. Following SDS-PAGE electrophoresis and PVDF membrane transfer, the membrane was cut perpendicular to the Protein Ladder. Each fragment of the membrane was blocked with 5% Bovine Serum Albumin (BSA) (one hour, room temp). Next, the membranes were incubated overnight at 4 °C with one of the following first primary antibodies: IKKα (MA5-16157), IKKβ (LF MA0192), IKKγ (PA5-27335), CK2α (LF MA0223), IĸBα (PA5-22120), IĸBβ (PA5-84615), p53 (PLA-0072), NFĸB (MA5-15563), phospho-NFĸB-Ser536 (SAB4504490), phospho-IĸBα-Ser32/36 (MA5-15224), Tubulin (PA5-29444), GAPDH (MA5-15738) or Histone H3 (PA5-16183). The next day, the membranes were washed in TBST and incubated with HRP conjugated secondary antibody (one hour, room temp). The proteins were identified by chemiluminescence (ChemiDoc, Bio-Rad Laboratories, Hercules, CA, USA). The optical density of the bands was analyzed using ImageLab software (Bio-Rad Bio-Rad Laboratories, Hercules, CA, USA). Each experiment was replicated three times.

### 4.7. Electrophoretic Mobility Shift Assay (EMSA)

The LightShift™ EMSA Optimization and Control Kit (Thermo Scientific, Waltham, MA, USA) was used to check the activity of NFĸB in the HTR8/SVneo cell nuclear fraction after stimulation. The nuclear extract (4 ug) was incubated in 1X Binding Buffer with poly(dIdC) and biotinylated at the 5′-end of the primers, including the region (underlined) recognized by the active form of NFĸB (5′-Biotin-AGTTGAGGGGACTTTCCCAGGC- 3′). The mixture was incubated for 20 min and subjected to electrophoresis on 6% retardation gel. It was then transferred to a nylon membrane. Next, the membrane was crosslinked with the DNA under UV, and treated with Blocking buffer. All further procedures were performed according to the manufacturers’ instructions. HeLa nuclear cell lysate after TNFα stimulation (Abcam, Cambridge, UK) was used as control, either with biotinylated primers (positive control) or a mix of biotinylated and non-biotinylated primers (negative control). Each experiment was replicated three times.

### 4.8. Duolink II PLA Technology Assay

Briefly, HTR8/SVneo cell suspension was seeded, 0.01 × 10^6^ cells per well, in FBS medium on the Coverglass TC-treated 8-well chambers (Eppendorf, Hamburg, Germany) and incubated in hypoxic or normoxic conditions for 24 h. Next, the stimulating medium was added. After 96-h incubation in stimulating medium, the cells were washed twice in dPBS and fixed in ice-cold methanol (15 min, room temp.). Following washing and permabilization in dPBS with 0.1%Triton X-100, the cells were incubated with Duolink Blocking Solution (one hour at 37 °C) in a humidity chamber. The cells were incubated in a mix of primary antibodies against RSK1 (PA5-29215) 1:300 and p53 (MA5-12557) 1:200 in Duolink Antibody Diluent overnight at 4°C. The next steps were performed according to the Duolink^®^ In Situ Red Starter Kit Mouse/Rabbit instructions (Sigma-Aldrich, St. Louis, MO, USA). The complex was visualised with a ZEISS Axio Vert.A1 TL/RL-LED fluorescence microscope (ZEISS, Jena, Germany). Each experiment was replicated three times.

### 4.9. Statistical Analyses

The Statistica v13 (Statsoft, Statsoft, Kraków, Poland) and GraphPad Prism 5.0 (GraphPad Software, USA) packages were used for statistical analyses. The normal distribution was determined by the Shapiro–Wilk test. Normally distributed data were analyzed by the Student’s *t*-test (for single comparison) or ANOVA with the Bonferroni post hoc test (for multiple comparison). The non-normally distributed data were analyzed by the Mann–Whitney U-test or Kruskal–Wallis with Dunn’s post hoc test. The results were considered as significant for *p* < 0.05.

## 5. Conclusions

Although the level and activity of NFκB are elevated in preeclampsia, its mode of activation remains unknown. In our in vitro model of trophoblast cells cultured in preeclamptic and normotensive environments, higher NFκB activity was observed in cells incubated with medium containing serum from preeclamptic women. Our findings indicate that neither the canonical, non-canonical or atypical activation pathways play a critical role in NFκB activation in preeclamptic conditions with hypoxia. In this environment, the inhibition of p53 by a targeted siRNA significantly reduced the nuclear fraction of the active form of NFκB i.e., NFκB Ser536. This suggests that the p53/RSK1 complex plays a key role in the activation of NFκB in preeclamptic conditions. The same mechanism might be actuated in preeclamptic placentas.

## Data Availability

All data generated or analyzed during this study are in the manuscript and in the supplementary materials.

## Supplementary Materials

The following are available online at https://www.mdpi.com/article/10.3390/ijms221910200/s1, Figure S1: Western blot results., Table S1: The clinical characteristics of the women from whom the blood samples were obtained for the stimulation of HTR8/SVneo cell line.
