# Supplementary material for: The Preeclamptic Environment Promotes the Activation of Transcription Factor Kappa B by P53/RSK1 Complex in a HTR8/SVneo Trophoblastic Cell Line
Source: Int J Mol Sci. 2021 Sep 22;22(19):10200. doi: 10.3390/ijms221910200 (PMC8508006; doi:10.3390/ijms221910200)
Supplement: Supplementary file 1 [file ijms-22-10200-s001.zip › ijms-1324903-supplementary.pdf]

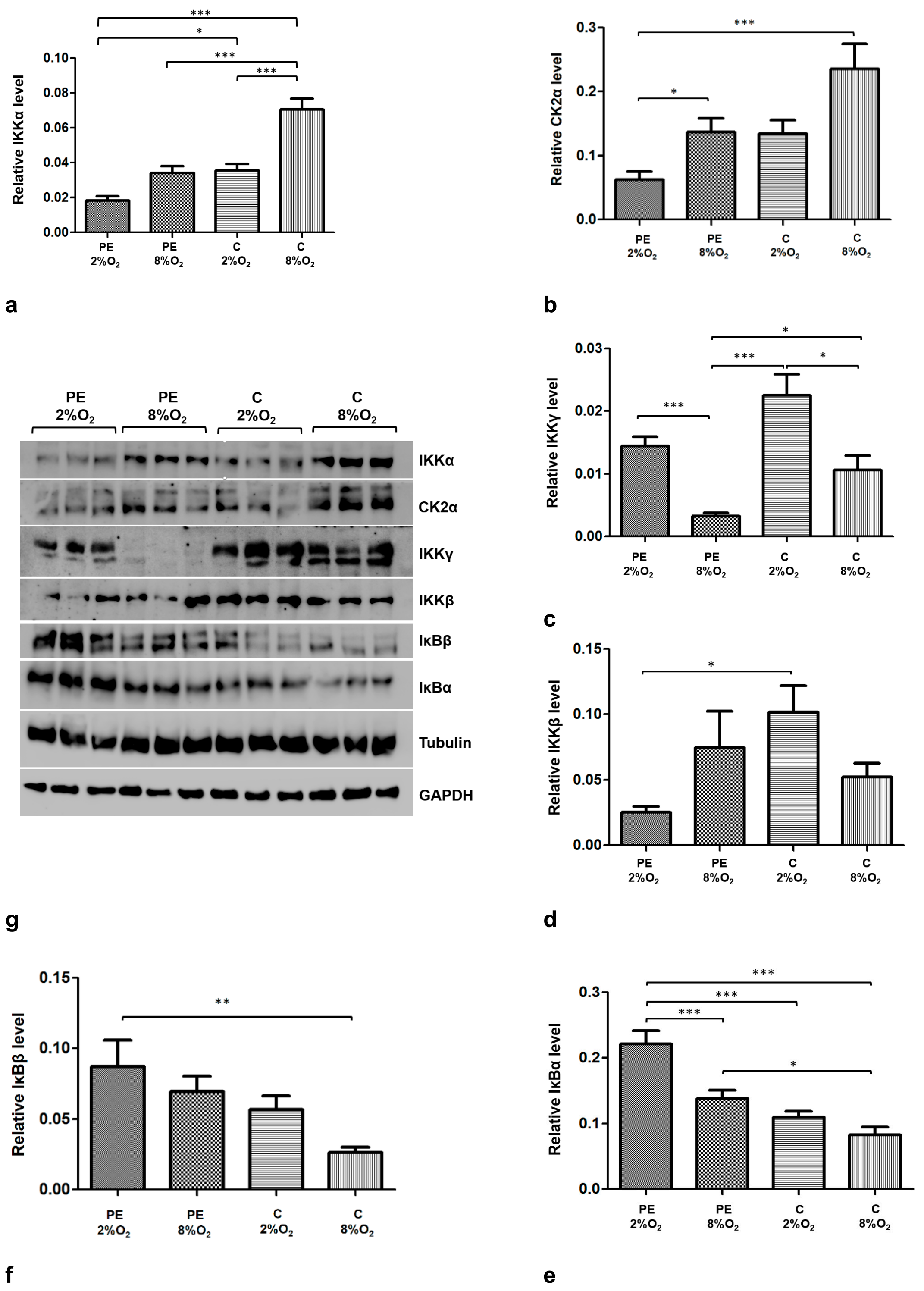

SI Figure S1 Western blot results. Comparison of relative levels of NFκB activators: IKKα (a), CK2α (b), IKKγ (c), IKKβ (d) and NFκB inhibitors: IκBα (e), IκBβ (f) between HTR8/SVneo cells cultured in hypoxia (2%O_2) and normoxia (8%O_2) in medium supplemented by 1% of serum from preeclamptic (PE) or normotensive (C) women. The relative levels were calculated to reference proteins: GAPDH and Tubulin. The Western blot membranes present the studied proteins (g). Data are presented as mean ± SEM, p-value calculated by ANOVA with the Bonferroni *post hoc* test or Kruskal-Wallis with Dunn's *post hoc* test, depending the data distribution.*p<0.05; **p<0.01, ***p<0.001

Table S1. The clinical characteristics of the women from whom the blood samples were obtained for the stimulation of HTR8/SVneo cell line

Parameters	Study group (preeclamptic pregnant women) N = 17	Control group (normotensive pregnant women) N=17	p
Maternal age at the time of delivery (years)[2]	32±4.2	33±4.5	0.559
WBC (10^3/ul)[1]	9.9 (6.9-14.3)	9.6 (8.0-14.8)	0.939
RBC (10^6/ul)[1]	4.1 (3.8-4.6)	4.1 (3.9-4.7)	0.756
HB (g/dl)[2]	12.3±1	12.4±1.2	0.811
HCT (%)[2]	35±2.4	35±2.8	0.845
MCV (fl)[2]	85.1±3.2	86.4±5.1	0.368
MCHC (g/dl)[1]	34.8 (32.7-36.0)	34.6 (33.3-36.1)	0.629
PLT (10^3/ul)[2]	193±56	221±55	0.156
BMI (kg/m^2)[2]	26±3	23±3	0.017
Newborn weight (g)[2]	2484±635	3214±395	0.003
Newborn length (cm)[2]	50±4	54±3	0.006
Week of delivery (week)[1]	37 (34-39)	38 (36-39)	0.117
Primiparous n (%)[3]	7 (41%)	3 (18%)	0.258
History of miscarriage n (%)[3]	4 (23%)	8 (47%)	0.282
Male sex of the fetus n (%)[3]	8 (47%)	7 (41%)	1.000

Legend: BMI, body mass index; WBC, white blood cells; RBC, red blood cells; HB, hemoglobin concentration; HCT, hematocrit; MCV, mean corpuscular volume; MCHC, mean corpuscular hemoglobin concentration; PLT, platelets; kg/m^2, kilograms/meter square; µl, microliter; g/dl, grams/deciliter; %, percent; fl, fentoliter; g, grams; cm, centimeter; n, number of cases; [1] non-normal distributed data presented as median and 10-90 percentile range, p value calculated by the Mann–Whitney U-test; [2] normal distributed data presented as mean±standard deviation, p-value calculated by Student's t-test; [3] categorical data, p value calculated by chi2 with Yates correction test. The p-value <0.05 was considered as significant for all tests.
